# Editorial: Metabolomics in genetic and endocrinological diseases

**DOI:** 10.3389/fmolb.2024.1365107

**Published:** 2024-01-19

**Authors:** Mohamed Abu-Farha, Hicham Benabdelkamel, Fabio Mazzotti, Anas M. Abdel Rahman

**Affiliations:** ^1^ Biochemistry and Molecular Biology Department, Dasman Diabetes Institute, Dasman, Kuwait; ^2^ Translational Research Department, Dasman Diabetes Institute, Dasman, Kuwait; ^3^ Proteomics Unit, Obesity Research Center, College of Medicine, King Saud University, Riyadh, Saudi Arabia; ^4^ Dipartimento di Chimica e Tecnologie Chimiche, Università Della Calabria, Rende, Italy; ^5^ Metabolomics Section, Department of Clinical Genomics, Center for Genome Medicine, King Faisal Specialist Hospital and Research Centre, Riyadh, Saudi Arabia; ^6^ Department of Biochemistry and Molecular Medicine, College of Medicine, Alfaisal University, Riyadh, Saudi Arabia

**Keywords:** metabolomics, DNA methylation, NAFLD, mass spectrometry, lipidomics

Metabolomics is an emerging technology for the global profiling of small molecules within a biological system. It has demonstrated significant potential in unravelling the mechanisms underlying various medical conditions, such as diabetes and cardiovascular diseases. Integrating metabolomics with other omics data, such as proteomics, transcriptomics, and genomics, facilitates the identification of novel pathways and disease mechanisms. The combination of metabolic biomarkers with established genetic and clinical variations holds promise for developing robust diagnostic and risk prediction models. Metabolites serve as a crucial link between genotype and phenotype, offering a valuable tool for stratifying patients with diverse clinical conditions. The availability of extensive genetic and phenotypic information from population genome projects, combined with the infrastructure of clinical genomics projects, has facilitated the integration of mass spectrometry (MS) and nuclear magnetic resonance (NMR) based metabolomics, contributing to improved health outcomes. In this Research Topic we covered multiple aspects of metabolomics such as integrating metabolomics data with DNA methylation as well as its utility in identifying unique metabolite signature for various diseases.

Torres et al., utilized and Integrated Metabolomics-DNA Methylation Analysis to study how exposure to proton irradiation (1 Gy of 150 MeV) affected the chemical makeup and DNA methylation in both the hippocampus and left ventricle of the heart in mice. The authors assessed both metabolomics and DNA methylation analysis 22 weeks after exposure proton irradiation. They showed significant changes in aminoacyl-tRNA biosynthesis, but the direction of these changes varied depending on the tissue. In the hearts of irradiated mice, the production of individual and total amino acids decreased, while it was increased in the hippocampus. Additionally, the authors were able to show that methylation-based epigenetic markers were modified, demonstrating that proton irradiation induces long-term changes in the heart and brain. Finally, the authors suggested that such a unique integrated approach could help identify biomarkers for radiation response, pinpoint therapeutic targets, and evaluate the effectiveness of interventions to minimize or prevent lasting damage from proton irradiation on the heart and brain.

Metabolomics has been extensively used for developing diagnostic and prediction models for various diseases disease. Multiple studies used either targeted or untargeted metabolomics for the identification of diagnostic diseases markers. Willems et al., evaluated the potential of plasma untargeted metabolomics using direct infusion high resolution mass spectrometry (DI-HRMS), in a clinical diagnostic setting for inherited metabolic diseases (IMDs) compared to targeted metabolomics. In a year-long pilot study involving 793 samples from patients diagnosed with IMD, the performance of DI-HRMS was compared with traditional targeted metabolite assays. The results demonstrated a strong correlation between concentrations measured in targeted assays and semi-quantitative Z-scores determined with DI-HRMS for the majority of metabolites. DI-HRMS detected aberrant metabolites indicative of IMDs in 55 out of 64 patients identified by targeted assays. Additionally, DI-HRMS revealed abnormalities in patients not detected by targeted plasma analysis, suggesting its potential as a first-tier approach in IMD diagnosis. The study concludes that DI-HRMS untargeted metabolomics could replace targeted assays and provides opportunities for discovering new biomarkers in the diagnosis of IMDs.

In another study Alfadda et al., focused on investigating changes in circulating lipid and metabolites involved in lipoprotein metabolism in patients with Type 2 diabetes mellitus (T2DM) with or without non-alcoholic fatty liver disease (NAFLD). The research included 434 T2DM patients where liver fat content was determined using FibroScan and lipidomic profile was assessed using High-throughput proton NMR. The authors showed a significant association between steatosis and increased concentrations of lipids, phospholipids, cholesterol, and triglycerides in very low-density lipoprotein (VLDL) and low-density lipoprotein (LDL) subclasses. On the other hand, high-density lipoprotein (HDL) metabolites were negatively associated with fatty liver contents. Moreover, liver was associated with elevated concentrations of lipids, phospholipids, cholesterol, and triglycerides in specific VLDL and HDL subclasses. Subgroup analysis indicated that patients using antilipemic medications exhibited a decrease in the concentrations of various lipid biomolecules.

Two other studies utilized metabolomics for identification of diagnostic diseases markers, the first one by Wang et al., where they investigated the association between metabolic disturbance and sudden sensorineural hearing loss (SSNHL). They animalized metabolites from 20 SSNHL patients and 20 healthy controls at baseline and after 3 months. Results revealed significant differences in metabolite signatures between SSNHL patients and healthy controls. The top 10 differential metabolites showed potential diagnostic value, with further analysis indicating that N4-acetylcytidine, p-phenylenediamine, sphingosine, glycero-3-phosphocholine, and nonadecanoic acid were predictors of hearing recovery. The study showed that distinctive serum metabolomics signatures in SSNHL patients and specific biomarkers hold promise for predicting hearing recovery and may contribute to understanding the underlying mechanisms and potential treatment options for SSNHL.

The second study by Chen et al., utilized metabolomics to identify metabolites in urine organic acids and characterize metabolic features for investigating the aetiology and pathogenesis of global developmental delay (GDD) and intellectual disability (ID). Positive test results explaining GDD/ID were screened from 1,253 cases. Among 1,230 cases with negative results (863 GDD cases, 367 ID cases), and 100 typically developing children (TD), non-supervisory principal component analysis and orthogonal partial least squares discriminant analysis were employed to distinguish GDD/ID from TD children and detect major differential metabolites. Twenty-three positive results explaining GDD/ID were obtained. Differential metabolites in the GDD and ID groups showed similar trends compared to the TD group, with 24 and 25 metabolites identified, respectively. These metabolites were associated with pathways such as ketone bodies synthesis and degradation, citrate cycle, alanine, aspartate and glutamate metabolism, pyrimidine metabolism, butanoate metabolism, pyruvate metabolism, fatty acid biosynthesis, valine, leucine, and isoleucine degradation. The study concluded that utilizing metabolomics to analyse urine organic acids in children with GDD/ID can reveal potential diagnostic biomarkers and contribute to understanding the conditions’ aetiology, pathogenesis, prognosis, and interventions. The last paper in this Research Topic by Guo et al., looked at exploring diagnostic biomarkers and underlying molecular mechanisms related to ferroptosis in Diabetic Nephropathy (DN).

In conclusion, this Research Topic explored the utilization of metabolomics technique in combination with other omics analysis or individually to explain diseases mechanism or for the identification of disease biomarkers.

